# Anévrismes de l'aorte abdominale sous-rénale rompus: aspects chirurgicaux à Dakar: à propos de 6 observations

**DOI:** 10.11604/pamj.2015.21.293.7637

**Published:** 2015-08-21

**Authors:** Papa Adama Dieng, Momar Sokhna Diop, Papa Salmane Ba, Souleymane Diatta, Magaye Gaye, Ndeye Fatou Sow, Papa Amath Diagne, Etienne Birame Sene, Amadou Gabriel Ciss, Assane Ndiaye, Mouhamadou Ndiaye

**Affiliations:** 1 Service de Chirurgie Cardiovasculaire et Thoracique, CHNU de Fann, Dakar, Sénégal

**Keywords:** Anévrysme aorte abdominale, rupture, chirurgie, Aneurysm abdominal aorta, rupture, surgery

## Abstract

La rupture des anévrismes de l'aorte est une situation catastrophique. La chirurgie ouverte et le traitement endovasculaire sont débattus comme option. Dans cette étude sont évaluées les modalités du traitement chirurgical et ses résultats sur 6 malades opérés pour rupture d'un anévrysme de l'aorte abdominale sous-rénale. Dans 4 cas, il s'agissait d'une « rupture contenue » dans le péritoine pariétal postérieur, avec un hématome péri vertébral. Dans les 2 autres cas, il s'agissait d'une rupture en péritoine libre. Tous les malades avaient bénéficié après contrôle de l'hémostase d'une mise à plat de l'anévrisme suivie de greffe d'une prothèse aortique. Des complications précoces étaient notées chez 2 patients à type d'insuffisance rénale aigue et de poussée hypertensive. La mortalité opératoire était de 16,66%. La morbi-motalité des anévrismes rompus de l'aorte abdominale est particulièrement élevée, d'où la nécessité d'un dépistage précoce des patients à risque et l'introduction de la chirurgie endovasculaire.

## Introduction

La rupture des anévrismes de l'aorte abdominale est une situation catastrophique avec une mortalité pré-hospitalière de 80% et une mortalité hospitalière de 30 à 60% [1]. Aux Etats-Unis, elle constitue la 13^ème^ cause de décès chez les personnes âgées de plus de 60 ans [2]. La meilleure stratégie thérapeutique en vue de réduire la mortalité reste débattue entre la chirurgie ouverte et le traitement endovasculaire. A Dakar, la chirurgie ouverte est la seule option utilisée. Le but de cette étude préliminaire était d’évaluer les modalités de ce traitement chirurgical et ses résultats.

## Méthodes

**Figure 1:**
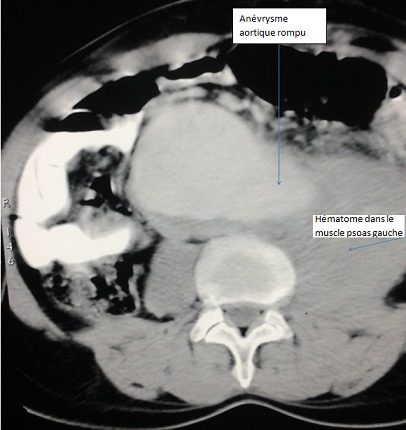
Scanner abdominal, coupe transversale

**Figure 2:**
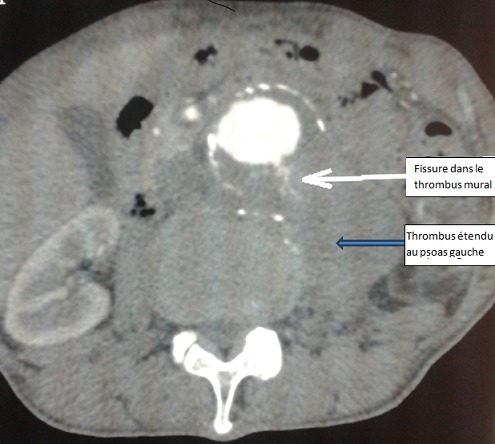
Scanner abdominal, coupe transversale

Il s'agissait d'une étude rétrospective descriptive allant de janvier 2004 à juillet 2013 portant sur 6 malades opérés pour rupture d'un anévrisme de l'aorte abdominale sous-rénale au Service de Chirurgie Cardiovasculaire et Thoracique du Centre Hospitalier Universitaire de Dakar. Tous les malades opérés étaient de sexe masculin. L’âge moyen était de 64 ans (58-73 ans). Cinq patients étaient originaires du Sénégal et un de la Guinée. Dans les facteurs de risque vasculaire, on retrouvait une hypertension artérielle chez 5 malades; qui évoluait en moyenne depuis 18 ans (3-26 ans). Une notion de tabagisme actif était également notée chez 5 patients (31 paquets-année en moyenne). La symptomatologie fonctionnelle était faite d'une douleur lombaire chez 4 patients, d'une douleur abdominale chez 3 patients, et de psoitis chez 2 patients. Deux malades étaient reçus en état de choc avec collapsus cardiovasculaire. L'examen physique retrouvait une masse abdominale pulsatile, battante dans 3 cas. L’échographie abdominale n’était réalisée que dans 3 cas et ne permettait de poser le diagnostic de rupture que dans 2 cas. Le scanner abdominal était réalisé dans tous les cas et montrait des signes de rupture dans la moitié des cas (Figure 1 et Figure 2). Le diamètre moyen de l'anévrisme était de 56 mm (47-65 mm). La morphologie du sac anévrismal était fusiforme dans 5 cas et sacciforme dans 1 cas. On notait une extension aux artères iliaques dans 1 cas. Les patients avaient bénéficié d'une réanimation préopératoire, avec remplissage par des macrolides, après mise en place d'une voie veineuse centrale et parfois d'une voie artérielle.

## Résultats

**Figure 3:**
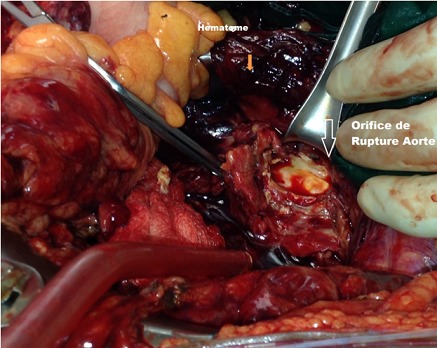
Vue opératoire de l'exposition de l'aorte abdominale

Tous les patients ont bénéficié d'une chirurgie ouverte en urgence. La voie d'abord était une laparotomie xipho-pubienne, sous anesthésie générale avec intubation orotrachéale. Le diagnostic de rupture était évoqué dans tous les cas en préopératoire grâce a l'examen clinique et paraclinique, et confirmé à l'exploration chirurgicale. Dans 4 cas, il s'agissait d'une « rupture contenue » dans le péritoine pariétal postérieur, avec un hématomepéri vertébral. Cet hématome était étendu au muscle psoas dans 2 cas (Figure 1). Dans les 2 autres cas, il s'agissait d'une rupture en péritoine libre, avec effusion de sang dans la cavité péritonéale. Un patient avait déjà présenté un arrêt cardiorespiratoire, récupéré en préopératoire, et le second avait présenté une rupture peropératoire secondaire d'un anévrisme thoracique, lors du clampage aortique abdominal. Il a succombé sur table opératoire malgré une réanimation et une transfusion sanguine importante. Un seul patient présentait une extension de anévrisme au niveau de l'artère iliaque commune gauche. L'exploration chirurgicale retrouvait l'orifice de rupture aortique et un hématome rétro péritonéal (Figure 3) ou du sang épanché dans la cavité péritonéale. La première étape chirurgicale consistait à un contrôle rapide de l'hémorragie, par clampage de l'aorte, sans perdre du temps dans une dissection minutieuse. Tous les malades avaient bénéficié d'une mise à plat de l'anévrisme avec greffe d'une prothèse de type Poly-tétra-fluoro-éthylène(PTFE) dans 5 cas, et dacron dans 1 cas. Dans 2 cas, il s'agissait d'un tube aortique et dans 4 cas d'une prothèse bifurquée aorto-bi-iliaque. La durée moyenne du clampage aortique était de 65 minutes(40-100 minutes). La fermeture était réalisée avec un drain dans le cul-de-sac de Douglas. Les pertes sanguines étaient estimées à 675 ml en moyenne. Trois patients avaient bénéficié d'une transfusion de sang total en pré et peropératoire (3 poches en moyenne). Des complications précoces étaient notées chez 2 patients, à type d'insuffisance rénale aiguë et de poussée hypertensive, qui ont prolongé le séjour en réanimation. Ainsi, la durée moyenne du séjour en réanimation était de 8 jours (1; 24 jours) et la durée moyenne du séjour en hospitalisation était de 16 jours (7; 25 jours). La mortalité opératoire concernait 1 cas, tandis que la mortalité précoce hospitalière concernait 2 cas. La durée de suivi était de 28 mois (1; 60). La mortalité tardive avait concerné un autre patient à 6 mois post-opératoires.

## Discussion

L'anévrisme de l'aorte abdominale est une pathologie complexe multifactorielle avec des facteurs génétiques et environnementaux. Il est beaucoup plus fréquent chez l'homme tandis que la femme présente un plus haut risque de rupture [3]. Dans notre étude on note une prédominance masculine comme dans la série de Kunishige et al [4] ou il y a 80% de patients de sexe masculin. Dans l’étude de Pryor [5], la douleur est le symptôme le plus fréquent (abdominale ou lombaire) comme dans notre série. Deux patients (le tiers de la série) étaient admis dans un tableau d’état de choc avec collapsus cardiovasculaire; nécessitant une stabilisation de l’état hémodynamique. Dans l’étude de Pryor [5], on retrouve 50% d’état de choc. Le scanner pose le diagnostic de rupture dans la moitié des cas. Ceci montre toute la difficulté de poser le diagnostic de rupture de façon précise, et de la prise en charge rapide de ces malades. Cette difficulté est notée dans l’étude de Greatorex et al [6], où le scanner pose le diagnostic de rupture dans 1 cas sur 6. Cependant, le contexte de survenue récente d'une douleur vive, un déséquilibre hémodynamique et des signes scannographiques, permettent à défaut de poser le diagnostic de certitude dans tous les cas, d'avoir une forte suspicion de rupture. Le diagnostic de certitude est fait en peropératoire. Il s'agit d'une « rupture contenue» dans la région rétro péritonéale ou d'une petite brèche « colmatée » par un hématome salvateur. Les patients avec une rupture cataclysmique en péritoine libre n'arrivent souvent pas vivants à l'hôpital. Le geste chirurgical commence par un contrôle de l'hémostase. Une mise à plat est ensuite réalisée, suivie d'une greffe aortique par une prothèse en tube ou bifurquée selon la topographie de anévrisme. Cette mise- à- plat- greffe est l'opération princeps décrite par De Bakey.

L’étude de Stollwerck et al [7] montre que les prothèses en PTFE, plus souvent utilisées dans notre pratique, présentent une plus grande résistance à long terme par rapport aux prothèses en polyester (dacron). Les pertes sanguines dans notre courte série sont de 675 ml en moyenne. Kunishige et al [4] ont montré que des pertes sanguines supérieures à 3000 ml sont significativement corrélées à une augmentation de la mortalité précoce, de même que la présence d'un état de choc en préopératoire. Dans notre série, 2 patients présentaient des complications précoces à type d'insuffisance rénale aiguë et de poussée hypertensive. Kunishige et al [4] retrouvent 8,7% d'insuffisance rénale en post-opératoire. L'insuffisance rénale aiguë est présente dans 2 à 10% des patients après la chirurgie ouverte pour anévrisme de l'aorte abdominale et le risque est augmenté avec l'hypoperfusion due au clampage aortique, l'hémorragie peropératoire et les emboles cruoriques [8]. La mortalité opératoire et périopéatoire dans cette étude est proche de celle rapportée parKunishige et al [4] qui est de 22,9%. Ce taux est acceptable, vu la gravité d'une rupture aortique et la complexité de sa prise en charge opératoire et post-opératoire.

## Conclusion

La rupture de anévrisme de l'aorte est une urgence majeure. Le diagnostic est évident quand elle se fait en péritoine libre, mais dans les cas de rupture contenue rétro-péritonéale, la certitude n'est obtenue qu’à l'exploration chirurgicale. Sa morbi-mortalité est très élevée. L'introduction des méthodes endovasculaires dans notre pratique, devrait permettre une meilleure prise en charge.
